# Flexible Resources Key to Neighborhood Resilience for Children: A Scoping Review

**DOI:** 10.3390/children10111791

**Published:** 2023-11-07

**Authors:** Shawna Beese, Kailie Drumm, Kayla Wells-Yoakum, Julie Postma, Janessa M. Graves

**Affiliations:** 1 College of Agricultural, Human, and Natural Resources Sciences (CAHNRS), Washington State University, Pullman, WA 99164, USA; kayla.wells@wsu.edu; 2 College of Nursing, Washington State University, Spokane, WA 99201, USA; jpostma@wsu.edu (J.P.); janessa.graves@wsu.edu (J.M.G.); 3 Nursing Program, Lower Columbia College, Longview, WA 98632, USA; kdrumm@lowercolumbia.edu

**Keywords:** neighborhoods, adverse childhood experiences, stress resilience

## Abstract

Neighborhoods have been the focus of health researchers seeking to develop upstream strategies to mitigate downstream disease development. In recent years, neighborhoods have become a primary target in efforts to promote health and resilience following deleterious social conditions such as the climate crisis, extreme weather events, the global pandemic, and supply chain disruptions. Children are often the most vulnerable populations after experiencing unexpected shocks. To examine and describe conceptually the construct of Neighborhood Resilience, we conducted a comprehensive scoping review using the terms (“resilience” or “resiliency” or “resilient”) AND (“neighborhood”), utilizing MEDLINE (through PubMed) and CINAHL (through EBSCOhost) databases, to assess overall neighborhood themes that impact resilience. A total of 57 articles were extracted that met inclusion criteria. Extracted characteristics included study purpose, country of origin, key findings, environmental protective/risk factors. The analysis revealed a positive relationship between neighborhood resource density, neighborhood resiliency, and individual resiliency. This study reports the finding for studies with a population focus of pre-school age and school age children (1.5–18 years of age). Broadly, we identified that the primary goals regarding neighborhood resilience for childhood can be conceptualized as all activities and resources that (a) prevent trauma during childhood development and/or (b) mitigate or heal childhood trauma once it has occurred. This goal conceptually encompasses antecedents that increase protective factors and reduces risk factors for children and their families. This comprehensive look at the literature showed that a neighborhood’s ability to build, promote, and maintain resiliency is often largely dependent on the flexible resources (i.e., knowledge, money, power, prestige, and beneficial social connections) that are available.

## 1. Introduction

Recent global events that have impacted the widespread childhood experience for children include the 2008 global financial crisis [1], COVID-19 pandemic [2], loss of land and ecosystems [3], increased vector-borne illnesses [4,5], and agricultural instability [6]. Children and adolescents are especially vulnerable to these stressors for a variety of reasons [7,8]. The ability of a child or adolescent to appropriately overcome the stressors surrounding them is in part dependent on the strength and ability of the systems in which they are a part [9]. This includes neighborhoods. Social determinants of health and mental health may now include factors such as socioeconomic status and resource availability [10]. For example, critical access to healthcare services is often limited in underserved or disadvantaged communities. Similarly, ecological determinants of health include clean water and food access, along with neighborhood and community abilities to overcome the consequences of natural disasters [11].

Neighborhoods are a promising setting to promote health and resilience [12,13]. Neighborhood disadvantages have been associated with an increased overall stress burden experienced by residents of those neighborhoods. Conversely, residence in affluent neighborhoods is associated with low to normal stress burdens [12]. This is an especially important consideration when working with children and adolescents after exogenous shocks are experienced [14,15]. The collective efficacy experienced at the neighborhood level may be key to building the resilience capacity of the children and families who live there [16]. Despite the need to address upstream factors that impact health, prevention programs too often focus on individual-level health behaviors and risk factors [17,18].

This scoping review aims to develop a clear conceptual definition of resilience in the context of neighborhoods and children. The secondary aim of this study is to identify key risk and protective factors to be considered when developing neighborhood-level pediatric health promotion strategies. To address these aims, we comprehensively reviewed the literature on resilience in the neighborhood setting.

## 2. Materials and Methods

Scoping reviews are preferable to systematic reviews when study objectives relate to the clarification of key concepts and the identification of dimensions related to a concept [19]. As such, due to the exploratory nature of this research inquiry, a scoping review was chosen as the methodology for synthesizing the literature. Utilizing the scoping review framework described by Arksey and O’Malley (2005), we (a) identified our research inquiry, (b) identified search parameters, (c) defined the study selection, (d) charted the data, and (e) collated, summarized, and reported the results [20].

### 2.1. Search Terms, Sources of Data, and Inclusion Criteria

On 5 July 2022, we conducted searches using the search terms [(“resilience” or “resiliency” or “resilient”) AND (“neighborhood”)], utilizing MEDLINE (through PubMed) and CINAHL (through EBSCOhost) databases. Inclusion criteria included English language publication in a peer-reviewed journal and data collection involving human subjects. Although our search protocol focused on the general concept of neighborhood resilience, studies reported in this review were limited to those related to a pediatric population (pre-school and school-age children, e.g., children aged 1.5–18 years old). Articles were included if pediatric population and resilience were addressed in the contexts of neighborhoods. No date range was defined for this study (all studies through 5 July 2022 were considered).

The concept of neighborhood was not predefined by the reviewer team. Of interest to us was how the construct of “neighborhood” was conceptualized by the authors of the reviewed articles and the populations being studied [21]. Results were imported into Covidence™ [22], a software program that facilitates synthesis reviews. PROSPERO does not register scoping reviews; consequently, this study was not registered. All study procedures adhered to the PRISMA-ScR checklist for scoping reviews [23]. 

As part of our a priori protocol, we included any study that provided insight into the antecedents, attributes, or consequences of neighborhood resilience. Quality appraisal on each article was not conducted due to the exploratory conceptual focus. Study characteristics that were extracted included authors, year of publication, country in which the research was conducted, title, study design method, study purpose, population, sample size, and key findings.

### 2.2. Data Analysis Process

Two authors (SB and KD) independently reviewed the articles to assess if the inclusion criteria were met and were blinded to voting until all reviews had been conducted. Voting and evaluation interrater reliability for each decision stage was carried out in Covidence™. Cohen’s Kappa was used to determine interrater reliability during the initial screening of title and abstract for inclusion/exclusion and full-text inclusion/exclusion phases. The authors reconciled any discrepancies in the inclusion and exclusion reviews through routine synchronous discussion via Zoom. Additionally, authors memo’ed and met routinely to discuss findings during the analysis and synthesis phase. Covidence™ software was used to standardize the extraction process conducted by SB, which was verified by KD and KWY. The results of the extraction process were exported for analysis and development (Table 1).

## 3. Results

**Figure 1:**
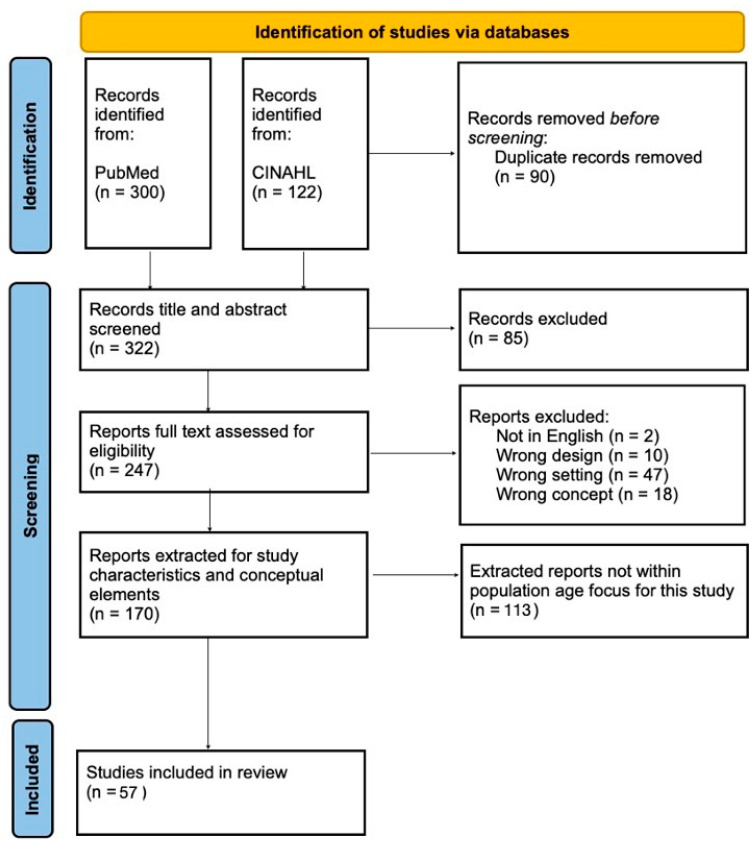
PRISMA flowchart of database search conducted on 5 July 2022 using the search terms. [(“resilience” or “resiliency” or “resilient”) AND (“neighborhood”)].

The initial search returned 422 articles with 90 duplications (Figure 1). Two authors independently screened the title and abstract of 332 articles and reviewed the full text of 247 articles. For the larger general search, which is beyond the scope of reporting in this manuscript, 170 articles met the inclusion criteria and were extracted for analysis. Included articles were published between 1994 and 2022. The primary reasons for excluding articles during the full-text review phase were because they did not focus on neighborhoods (N = 47) or resilience (N = 18). Articles were excluded if they were not a study (N = 10) or published in English (N = 2). All 170 articles were extracted for our larger neighborhood resilience analysis.

To narrow the articles being reported for this manuscript, only 57 studies were retained for the narrowed inclusion criteria focusing on the pediatric population and pre-school age and school-age children (ages 1.5–18 years old).

### 3.1. Broad Themes

There was fair agreement between the reviewers for the title/abstract screening phase (Cohen’s κ = 0.39) and moderate agreement for the full-text reviews (κ = 0.47) between independent raters [81]. Broadly, the results suggest that neighborhood resilience for childhood can be conceptualized as all activities and attributes that (a) prevent trauma during childhood development and/or (b) mitigate or heal childhood trauma once it has occurred. This conceptually encompasses antecedents that increase protective factors and reduce risk factors for children and their families.

Authors of articles included in this study describe “skin-deep” resilience as the against-all-odds ability to rise above the traumas one has experienced in childhood [33]. Skin-deep resilience speaks to the phenomena where children of disadvantaged neighborhoods, through individual attributes such as conscientiousness and hard work, achieve external success such as high educational attainment or upward social economic mobility [82,83]. The individual attributes of high conscientiousness and productivity in work are correlated with less substance use and increased outward achievement, but these children also experience a higher prevalence of health conditions associated with cumulative “weathering” stress. Brody and Chen’s work is primarily focused on African–American youth. However, the weathering effect has also been noted in children who have experienced adverse childhood experiences (ACEs) [84].

This concept of overcoming past trauma was addressed in the multiple studies that examined adverse childhood experiences (ACEs) [34,36,37,41,42,44,52,55,62,78,80]. In more recent years, the literature has cautioned that framing resilience as an individual attribute can re-victimize children who have been abused with the further critique that they lack the qualities needed to overcome their trauma. We identified studies on brain function as noteworthy [68], although, theoretically, we focused on studies regarding neighborhood and familial characteristics [29,30,31,32,43,47,49,50,53,56,70,71,74,75,77]. One study explored how an increase in resources contributes to positive childhood events (PCEs) [40].

We contrasted skin-deep resilience that is based on the attributes of an individual child and the potential of a deeper-rooted resilience that is supported across all the nested systems of a neighborhood. Two studies focused on environments that promote flourishing beyond the baseline and where access to flexible resources across all levels is crucial [28,58]. In these studies, the protective resilience that is provided through positive childhood experiences such as having a mentor, family resiliency, and networked community connections is discussed. Creating protective resilience was central to Breton’s (2001) work on neighborhood resilience. Breton posited that what makes a neighborhood resilient is social and physical resources that improve one’s ability to adapt. Initial resource availability and a state of equilibrium must be present, such that when disequilibrium occurs, whether by a disaster or similar event, resources may be tapped into to reinstate the equilibrium of the neighborhood [85]. Breton argues that this ability is dependent on resources and infrastructure and that policies that increase these resources must be protected.

After a review of the literature, we define the construct of neighborhood resilience as “the protective capacity of a neighborhood to restore itself, and promote adaption among citizens, after experiencing an adverse exogenous shock” [26,85]. The conceptualization of resilience and level of resilience addressed by reviewed articles can be found in Supplementary Table S1.

To aide conceptual exploration and refinement, notes on risk and protective factors are reported in Table 1. The findings can be thematically categorized into two groups: neighborhood advantages that exhibit a positive association with resilience and neighborhood disadvantages that demonstrate a negative association with resilience.

### 3.2. Neighborhood Advantages

Neighborhood advantages are all the protective factors that increase resilience for the citizens who live there. Within the context of promoting resilience among children, community connections emerged as a significant neighborhood advantage. Constructs such as neighborhood cohesion, collective efficacy, and neighborhood connectedness were identified as key factors. Neighborhood cohesion is defined as the degree to which neighbors are interconnected and supportive [64]. In neighborhoods with high collective efficacy, residents are more likely to take collective action on behalf of each other and the neighborhood as a whole [86,87]. Furthermore, neighborhood connectedness encompasses both the quality and quantity of relationships maintained among neighbors.

Predictably, neighborhoods that demonstrate collective efforts to increase resilience capacity and proactively prepare for the unforeseen serve as protective environments for children and families residing within them. Planning activities include assessing neighborhood resources and the availability of neighborhood services [88]. Other studies focused on developing a community understanding of resilience and how communities can prepare for future disasters [35,89] or hasten recovery post-disaster [90].

### 3.3. Neighborhood Disadvantages

Neighborhood disadvantages were conceptualized in this analysis as all risk factors that decreased the capacity for resilience and exhausted any banked capacity among citizens. The presence of disorder and perceived senses of being unsafe within a neighborhood are strongly associated with a decreased capacity for resilience. A primary goal in promoting resilience across the life-course is the prevention of childhood trauma. Neighborhood disorder and incivilities experienced in neighborhood spaces are clearly identified risk factors. Studies have consistently demonstrated that exposure to neighborhood violence [24] and perceived neighborhood disorder are associated with a decreased resilience capacity [48,69]. Similarly, perceived neighborhood incivilities decrease a neighborhood’s capacity for resilience. These incivilities include nuisance crimes [45,46].

## 4. Discussion

This study cataloged the multi-layered aspects of what makes a neighborhood resilient and how that resiliency can affect outcomes in children and adolescents. The analyzed studies highlight the effects of neighborhood stability and resource availability on individual health, wherein increased exposure to stressors and instability are linked to increased cumulative stress and chronic disease development. Given the significant influence of neighborhoods on health and wellness, it is imperative that neighborhood citizens recognize that an erosion of social capital occurs as resource availability is diminished [91]. Yet there are many neighborhood-level actions that can be taken that are not resource intensive but rather rely upon social capital and connectedness. Simple activities that engage neighbors in building social supports for each other and particularly for the children of the neighborhood will likely improve a sense of connectedness. One potential neighborhood action would be to collectively identify the risk factors and protective factors in their neighborhood. Once a consensus is built around identification, engaging in the superordinate goal of increasing the protective factors (flexible resources) in the neighborhood or mitigating risk would likely increase neighborhood resilience.

As a midrange theory, fundamental cause theory could explain this relationship between higher socioeconomic status neighborhoods wherein increased resources allowed for more resiliency [92]. In contrast, individuals residing in lower socioeconomic status neighborhoods face a disadvantage due to limited access to these flexible resources [93,94]. If basic needs are not being met for neighborhood citizens, no amount of creativity will convert absolute deprivation into flexible resources. However, building the capacity for resilience within a neighborhood could come in the form of creatively increasing the flexibility of the limited resources that are present. As such, neighborhoods with highly connected citizens will have an increased resilience capacity. Conversely, disadvantaged neighborhoods are more at risk for destabilization, possibly due to a lack of built social capital [95].

Children and adolescents are psychologically vulnerable. Developmental psychopathology frameworks explain that various stressors, including environmental, neurobiological, and emotional stressors, can increase the prevalence of adverse mental health outcomes based on developmental staging [96]. This review reinforces the positive relationship between increased access to flexible resources such as beneficial social connections, knowledge, money, power, and prestige, and increased neighborhood adaptive capacity. Favorable social conditions with flexible resources promote increased adaption to unforeseen events of all types [97].

### Strengths and Limitations

A limitation of this study was the variability of independent reviewer voting as manifested by fair to moderate interrater reliability scores. This scoping review was exploratory by nature. Achieving an eventual consensus between two independent reviewers through memo’ing and discussion clarified the conclusions drawn.

An additional limitation was that we did not conduct quality appraisals on each article. We established an a priori protocol for our study and mitigated potential bias originating from our study. However, inclusion criteria for this study were based on the potential conceptual value and not the rigor of the reviewed study. Although all the included studies had conceptual value for the discourse of neighborhood resilience, not all reviewed studies were of quality or designed to, on their own, inform practice changes.

A strength of this scoping review is the comprehensiveness of the literature reviewed. Our specific objective was to review articles that contribute to the conceptual understanding of neighborhood resilience across all contexts found in the literature. As a result of the broad exploration undertaken by this study, the findings are presented in a concise and condensed manner, with greater emphasis on the wide breadth of literature than on detailed analysis. It is important to note that the intention of scoping reviews is to provide a broad assessment of the literature on a given topic and inform next steps, which we have effectively accomplished in this study.

## 5. Conclusions

With this study, we reviewed 28 years of literature and cataloged the risk factors and, perhaps more importantly, the protective factors of neighborhood resiliency. We have established the foundation on which our Extension community resource publications can be built upon. This scoping review contributes to the growing body of evidence regarding how neighborhood environmental factors influence the resilience of groups and individuals. This scoping review presents a foundation of how neighborhoods influence childhood resilience. Implications of this knowledge can help inform neighborhood health promotion strategies intended to build protective and resilient environments for children.

We recommend the next steps of this synthesized knowledge to be the future conceptual modeling of the core constructs of resilience in the neighborhood context. This scoping review can also contribute to discourse regarding the planning and policy development that directly affect resource availability in neighborhoods and, by proxy, promote positive health outcomes for youth.

## Figures and Tables

**Table 1:** Study characteristics for full-text reviews from database search conducted on 5 July 2022 using the search terms [(“resilience” or “resiliency” or “resilient”) AND (“neighborhood”)].

Author Year of Publication Country	Title	Methods	Purpose of Study	Study Population Sample Size	Key Findings	Protective and Risk Factors
[24] 2008 United States	Community violence in context: risk and resilience in children and families	Narrative review	The purpose of this study was to examine violence and neighborhood structure.	Neighborhoods (unspecified) N/A	A multi-level approach (beyond the individual) is required to address neighborhood violence. Socio-ecological factors have greater influence than individual resilience factors. This study was a narrative review of literature from the 1990s to the early 2000s but did not synthesize findings in a discussion session.	Risk Factors: Exposure to violence Neighborhood/community economic disadvantage Protective Factors: Parental support (family cohesion vs. parental empowerment of children) Adult mentoring Positive community organizations Perceived school/neighborhood safety Social capital
[25] 2008 United States	Cluster profiles of youths living in urban poverty: factors affecting risk and resilience	Cross-sectional	The purpose of this study was to examine risk and protective factors among the study population.	Urban youth N = 157	Cluster groups were labeled based on risk and protection factors.	Risk Factors: Poor supervision Poor discipline Delinquency Negative coping skills - disconnection from peers and isolation - drug use - low self-esteem Protective Factors: Social support School commitment/good grades Neighborhood cohesion Daily hassles Problems with peers
[26] 2018 United States	Use of theory to examine health responsibility in urban adolescents	Cross-sectional	The purpose of this study was to test relationships between resilience and variables of interest.	Urban adolescents N = 122	A significant relationship was found between health responsibility and health-promoting behaviors neighborhood perception No relationship was found between health responsibility and resilience social support	N/A
[27] 2016 United States	Cultural factors moderating links between neighborhood disadvantage and parenting and coparenting among Mexican-origin families	Cross-sectional	The purpose of this study was to explore the relationship between supportive parenting and neighborhood disadvantage.	Mexican-origin mothers N = 71	Familism beliefs support resilient parenting despite neighborhood disadvantage.	Risk Factors: Ecological stressors
[28] 2022 United States	Supportive neighborhoods, family resilience, and flourishing in childhood and adolescence	Cross-sectional	The purpose of this study was to examine protective factors among children and adolescents.	Children and adolescents N = 43,213 (18,396 children and 24,817 adolescents)	Direct association between higher levels of neighborhood social cohesion and higher levels of flourishing adolescents.	Protective factors: Supportive families
[29] 2021 United States	Deconstructing the role of the exposome in youth suicidal ideation: trauma, neighborhood environment, and developmental and gender effects	Cross-sectional	The purpose of this study was to evaluate the interaction between individual-level adversity and neighborhood environment and the effects on youth suicidal ideation (SI).	Children N = 7054	Assaultive trauma was strongly associated with SI.	Risk factors: Assaultive trauma
[30] 2022 United States	Social and relational health risks and common mental health problems among US children: the mitigating role of family resilience and connection to promote positive socioemotional and school-related outcomes	Cross-sectional	The purpose of this study was to explore social determinants of health among the study population.	US children N = 131,774	Greater family resilience and parent–child connection for children with MEB resulted in the youth demonstrating good self-regulation.	N/A
[31] 2021 United States	Protective factors to promote health and flourishing in Black youth exposed to parental incarceration	Cross-sectional	The purpose of this study was to examine the protective factors of the study population.	Black youth with incarcerated parent(s) N = 839	Family connectedness corresponded with good health and flourishing for Black youth exposed to parental incarceration.	Protective factors: Neighborhood connectedness
[32] 2018 United States	More than gangsters and girl scouts: environmental health perspectives of urban youth	Qualitative data analysis	The purpose of this study was to explore how the study population interpreted neighborhood health.	Urban youth of color N = 64	Focus group results were organized into two environmental health categories: Definitions: environmental health is multidimensional and integrative Concepts: (a) resilience factors such as safety and trust and (b) underlying structural drivers, such as power	Protective factors: Safety Trust Engagement Leadership Representation
[33] 2013 United States	Resilience to adversity and the early origins of disease	Literature review	The purpose of this study was to describe the expansion of research regarding health and the study population.	African–American rural youth Unspecified	Skin-deep resilience studies showed that some psychosocially resilient youth demonstrated high allostatic loads and accelerated epigenetic aging.	Risk factors: Economic hardship Downward mobility Neighborhood poverty Racial discrimination
[34] 2015 United States	The impact of neighborhood disorganization on neighborhood exposure to violence, trauma symptoms, and social relationships among at-risk youth	Cross-sectional	The purpose of this study was to examine the impact of neighborhood environment and violence.	Urban and rural Youth N = 2242	The findings emphasize the importance of providing trauma-informed care and maintaining social relationships.	Protective Factors: Youth social support Risk Factors: Exposure to violence (ETV)
[35] 2012 United States	Social resources and community resilience in the wake of superstorm Sandy	Cross-sectional	The purpose of this study was to examine relationships between neighborhood characteristics, family, and adolescent competence.	Neighborhood residents impacted by superstorm Sandy N = 1009	Identified protective factors are associated with higher levels of neighborhood preparedness.	Protective factors: Collective efficacy Social cohesion Social exchange
[36] 2019 United States	Validation of the traumatic events screening inventory for ACEs	Cross-sectional	The purpose of this study was to explore an adapted adverse childhood experience (ACE) screening tool.	Children N = 261	The adapted TESI is a valid tool for screening for ACEs.	Risk factors: Adverse childhood experiences (ACEs), especially ACEs among poly-victimized youth
[37] 2021 United States	Neighborhood disadvantage, childhood adversity, bullying victimization, and adolescent depression: a multiple mediational analysis	Cross-sectional	The purpose of this study was to examine the association among neighborhood health, adverse childhood experiences (ACEs), bullying, and depression.	Child/parent dyads N = 4898	Collective efficacy and disadvantaged neighborhoods directly impact ACEs, bullying, and social-emotional development.	N/A
[38] 2022 South Africa	Where you live matters: township neighborhood factors important to resilience among South African children from birth to 5 years of age	Cohort study	The purpose of this study was to examine whether neighborhood factors predicted child resilience in the study population.	South African children N = 1238	Resilient children in the high prevalence (resilience) neighborhoods were more food secure and their mothers were less depressed. Children high prevalence neighborhoods also migrated to rural areas more often.	N/A
[39] 2015 Australia	Maternal efficacy and sedentary behavior rules predict child obesity resilience	Cohort study	The purpose of this study was to identify if resistance to unhealthy weight gain has environmental predictors.	Children N = 200	Setting rules to limit sedentary behaviors predicted child resiliency to unhealthy weight gain.	Protective factors: Neighborhood safety (physical safety as well as neighborhood network) Neighborhood play spaces Neighborhood access to quality food
[40] 2021 United States	Positive childhood experiences promote school success	Cross-sectional	The purpose of this study was to test relationships between positive childhood experiences (PCEs) and school success.	Youth N = 33,450	Most cited PCEs included having a mentor, family resiliency, and participation in after-school activities. Children living in supportive neighborhoods had fewer reported school absenteeism.	Protective factors: Educational attainment Family resilience Mentor After-school participation Caregiver to share ideas with Supportive neighborhood
[41] 2022 United States	Examining the influence of positive childhood experiences on childhood overweight and obesity using a national sample	Cross-sectional	The purpose of this study was to examine associations between positive childhood experiences (PCEs) and obesity among the study population.	Children N = 28,771	For children who experience trauma, PCEs may mitigate obesity.	Protective factors: Safe and supportive neighborhoods
[42] 2018 United Kingdom	Protective factors for psychotic symptoms among poly-victimized children	Cohort study	The purpose of this study was to explore associations between the development of psychotic symptoms and polyvictimization in the study population.	Twin children N = 2232	The following are protective factors that could mitigate childhood poly-victimization: High IQ Positive home atmosphere Higher levels of neighborhood social cohesion.	Risk factors: Childhood trauma
[43] 2019 United States	Two-year changes in neighborhood juvenile arrests after the implementation of a park-based afterschool mental health promotion program in Miami–Dade County, Florida, 2015–2017	Cohort study	The purpose of this study was to examine associations between mental health programs and neighborhood resilience.	Children N = 501	Park-based programs targeting at-risk youth may promote mental health and resilience and prevent violence.	N/A
[44] 2000 United States	Resilient and stress-affected adolescents in an urban setting	Cross-sectional	The purpose of this study was to test the relationship between stress and material protective factors.	Urban adolescents N = 185	No relation between stress and material protection for individually resilient adolescents.	N/A
[45] 2009 United States	School engagement among urban adolescents of color: does the perception of social support and neighborhood safety really matter?	Cross-sectional	The purpose of this study was to examine the relationship of risk and protective factors to school engagement.	Seventh- and eighth-grade students N = 123	Social support variables did not lessen the effects of risky neighborhood conditions.	Risk factors: Not enough recreational facilities Lack of supervised activities Lacking key services such as police and trash collection
[46] 2020 United States	Safe spaces embedded in dangerous contexts: how Chicago youth navigate daily life and demonstrate resilience in high-crime neighborhoods	Qualitative data Analysis	The purpose of this study was to explore the safety strategies and resiliency of youth in high-crime neighborhoods.	Youth N = 15	Youth employees varied safety strategies which included identifying safe spaces in danger zones, hypervigilance, emotional management, and self-defense.	N/A
[47] 2020 United States	Mexican American urban youth perspectives on neighborhood stressors, psychosocial difficulties, and coping: en sus propias palabras	Qualitative data analysis	The purpose of this study was to explore urban youths’ perspectives on neighborhood stressors.	Mexican American youth N = 32	Pervasive stress was consistent across all groups, but coping styles and psychological difficulties varied. Themes related to stress include violence and poverty.	Protective factors: Trusted adults Positive peer mentoring group
[48] 2016 Cali, Columbia	A spatial model of socioeconomic and environmental determinants of dengue fever in Cali, Colombia	Spatial modeling	The purpose of this study was to examine environmental factors associated with rates of reported dengue fever.	Geographic regions N/A	Allocation (local) of resources and the better detection of high-risk areas will strengthen resilience in the local population.	Risk factors: Neighborhood disorder and filth Protective factors: Local planning
[49] 2018 United States	Resilience in Urban African American Adolescents: The protective enhancing effects of neighborhood, family, and school cohesion following violence exposure	Cross-sectional	The purpose of this study was to examine contextual influences of sense of self, belonging, and mood in relation to the proximity of violence for the study population.	Black American urban adolescents N = 269	Positive outcomes were directly related to neighborhood cohesion, as well as family cohesion.	Protective factors: Family and neighborhood cohesion
[50] 2021 United States	Promoting healthy trajectories for urban middle school youth through county-funded, parks-based, after-school programming	Non-randomized experimental study	The purpose of this study was to examine the effectiveness of promoting healthy processes to the study population.	Neighborhood parks and middle school youth N = 9 parks (198 youth)	There are promising results to support that sporting programs mitigate risks to resilience.	N/A
[51] 2022 United States	Disparity in the built environment and its impacts on youths’ physical activity behaviors during COVID-19 pandemic restrictions	Cross-sectional	The purpose of this study was to examine the relationship between neighborhood environments and the physical activity of the study population as a result of COVID-19 restrictions.	Children and adolescents N = 1324	Playing behaviors significantly increased: In yards In neighborhoods Playing behaviors significantly decreased: In community-based play	N/A
[52] 2018 United States	Childhood adversity and parent perceptions of child resilience	Cross-sectional	The purpose of this study was to examine adverse childhood experiences (ACEs) and children’s resilience.	Children N = 62,200	Child resilience in relationship to ACEs was dose-dependent: as ACEs increased, resilience decreased.	N/A
[53] 1999 United States	Resiliency factors protecting against teenage alcohol use and smoking: influences of religion, religious involvement and values, and ethnicity in the Missouri adolescent female twin study	Cohort Study	The purpose of this study was to explore effects of ethnicity and religion on substance use in the study population.	Racial and ethnic minority female twins N = 474 (220 complete pairs)	Adolescent religious involvement and values are protective and contribute to lower rates of African–American alcohol use.	Protective factors: Adolescent religious involvement and values
[54] 2018 United States	Parenting behaviors, neighborhood quality, and substance use in 9th- and 10^th^-grade Latino males	Cross-sectional	The purpose of this study was to examine the association between neighborhood environment and parenting behaviors in relation to substance use in the study population.	Latino male 9th and 10th graders N = 379	Key findings include identifying risk and protective factors.	Protective factors: Neighborhood quality Father psychological control (only with high neighborhood quality) Risk factors: Mother psychological control (only with low neighborhood quality)
[55] 2018 United States	Promoting the development of resilient academic functioning in maltreated children	Cross-sectional	The purpose of this study was to examine the variations and extent of protective factors for language development and academic success among children who have been abused.	Children and adolescents N = 1776	Decreased language development and less academic success were associated with childhood neglect during infancy/toddlerhood or physical abuse during preschool age.	Protective factors: Child prosocial skills Caregiver warmth Caregiver cognitive stimulation
[56] 2014 United States	Exploring linkages between school climate, behavioral norms, social supports, and academic success	Exploratory factor analysis	The purpose of this study was to explore relationships between households and neighborhood environments and academic success in the study population.	Middle school students N = 13,068	Supportive relationships and norms promoting safe, prosocial behavior led to students having better grades and behavior in school.	Risk factors: Unsafe neighborhoods
[57] 2020 United States	Positive youth development in the context of household member contact with the criminal justice system	Cross-sectional	The purpose of this study was to explore protective factors that are associated with positive youth development (PYD).	Metropolitan youth N ≈ 2774	Results suggest that positive youth development (PYD) is associated with parental support and community belonging and connectedess.	Protective factors: Positive youth development- Maternal warmth Parental monitoring Religious activities Belonging at school Neighborhood belonging
[58] 2018 United States	Parental perception of flourishing in school-aged children: 2011–2012 National Survey of Children’s Health	Cross-sectional	The purpose of this study was to examine factors associated with flourishing in the study population.	Children N = 559,362	Factors significantly associated with flourishing: Parent education Age of child Physical activity Adequate sleep Family meals Extracurricular activities School safety Neighborhood safety and support No significance shown between flourishing and the below: Level of poverty Structure of household	N/A
[59] 2004 United States	Risk and resilience in urban children with asthma: a conceptual model and exploratory study	Exploratory data analysis	The purpose of this study was to define and model the concept of asthma-related resilience for the study population.	Children and primary caregivers N = 31	After neighborhood disadvantage and asthma symptoms were held constant, higher levels of adaptability acted as a resource factor. For children living with higher levels of neighborhood disadvantage, higher adaptability was shown to be a protective factor for increasing asthma management behaviors.	Risk factors: Neighborhood disadvantage Protective factors: Adaptable resource
[60] 2021 United States	Cumulative exposure to neighborhood conditions and substance use initiation among low-income Latinx and African–American adolescents	Cross-sectional	The purpose of this study was to identify and describe the substance use of the study population and neighborhood risks.	Latinx and African–American adolescents N = 736	Exposure to neighborhood social disorder shown to be a significant risk factor for the initiation of cigarette use.	Protective factors: Social capital Risk factors: Social disorder
[61] 1994 United States	Substance abuse among inner-city Hispanic women: exploring resiliency	Qualitative data Analysis	The purpose of this study was to identify factors that prevented drug abuse in the study population and identify preventative strategies.	Hispanic women N = 24	Inner-city Latina women in this study identified environmental attributes that increase resiliency in children.	Protective factors: Caring and support High expectations Opportunities to participate
[62] 2021 United States	Community-wide resilience mitigates adverse childhood experiences on adult and youth health, school/work, and problem behaviors	Cross-sectional	The purpose of this study was to examine if community-wide levels of resilience lessened the impact of adverse childhood experiences (ACEs) on the study population.	Youth N = 118	Both contextual and individual resilience mitigates ACE outcomes for adults; only contextual resilience mitigates ACE outcomes for youth. Contextual resilience: Factors of social capital Social cohesion Adult collective efficacy Protective support for youth	Protective factors: Supports for youth in four domains Family/adult Peer School Neighborhood/community
[63] 2018 United States	The impact of positive contextual factors on the association between adverse family experiences and obesity in a national survey of children	Cross-sectional	The purpose of this study was to evaluate the impacts of adverse family experiences (AFEs) on the study population becoming overweight or obese.	Children N = 43,864	Children exposed to one or more AFEs who also lacked resilience were at risk of becoming overweight and obese.	N/A
[64] 2022 United States	Informal supports, housing insecurity, and adolescent outcomes: implications for promoting resilience	Cohort study	The purpose of this study was to examine associations between adolescent behavior, neighborhood cohesion, and housing insecurity.	Families N = 2425	Neighborhood cohesion in childhood is a protective factor for adolescent aggressive behaviors. Additionally, informal supports provide resilience for low-income families.	Protective factors: Neighborhood cohesion Instrumental support in childhood
[65] 2007 United States	Risk and protective factors predictive of sense of coherence during adolescence	Cross-sectional	The purpose of this study was to identify risk and protective factors across adolescent ecology that predict sense of coherence (SOC), as well as to explore gender differences in the study population.	Middle school students N = 1619	Predictors of SOC for both genders: Social support Anger expression Family conflict Neighborhood cohesion	Protective factors: Social support and neighborhood cohesion
[66] 2021 United States	Resilience to COVID-19: socioeconomic disadvantage associated with higher positive parent–youth communication and youth disease prevention behavior	Cohort study	The purpose of this study was to explore neighborhood environment and the disease burden of COVID-19.	Metropolitan youth N = 6874	Parents’ reports of higher family COVID-19 exposure risk and diagnosis were associated with both family and neighborhood disadvantage.	Protective factors: Open parent–youth communication
[67] 2021 United States	Surviving all the way to college: pathways out of one of America’s most crime-ridden cities	Qualitative data Analysis	The purpose of this study was to explore academic failure and involvement in the criminal justice system for the study population.	Students N = 146	The protective factors trifecta confirms previous studies that indicate that resilience is not determined by individual traits of youth, but rather environmental exposures of youth.	Protective factors: Engaged parenting Self-selected high schools and the interaction of an individual’s inner traits Local ecological supports
[68] 2021 United States	Resting-state functional connectivity of the central executive network moderates the relationship between neighborhood violence and proinflammatory phenotypes in children	Cross-sectional	The purpose of this study was to examine the relationship between the neighborhood environment and phenotypes in the study population.	Children N = 217	The central executive network moderates the relationship between neighborhood violence and inflammation in children.	Risk factor process: Trauma from neighborhood violence ---> inflammatory response [moderated by resting-state functional connectivity (rsFC) of the central executive network (CEN)] ---> future adverse outcomes secondary to stress
[69] 1999 United States	Perceived crime and informal social control in the neighborhood as a context for adolescent behavior: a risk for resilience perspective	Cross-sectional	The purpose of this study was to explore relationships between adolescent perception of neighborhood crime, social control (informal), and behavior problems.	Middle and high school students N = 2099	Adolescent perception of neighborhood crime and behavior problems critically influence social context, this is particularly true in disorganized neighborhoods.	Risk factors: Normalizing social disorganization elements such as drugs, gangs, and violence
[70] 2021 United States	Hispanic parents’ views of family physical activity: results from a multisite focus group investigation	Cross-sectional	The purpose of this study was to evaluate parental views on the physical activity of children and family.	Mexican–American and Puerto Rican parents N = 61	Major obstacles to family physical activity: (a) time constraints, (b) unsafe neighborhood streets, and (c) unsafe neighborhood parks.	N/A
[71] 2022 United States	School health predictors of the school-to-prison pipeline: substance use and developmental risk and resilience factors	Cross-sectional	The purpose of this study was to explore potential relationships between substance use, risk/resilience factors, and school discipline.	Students N = 4,950,000	Increased community support and student safety are associated with less school discipline/police.	N/A
[72] 2016 United States	Youth withdrawal moderates the relationships between neighborhood factors and internalizing symptoms in adolescence	Cross-sectional	The purpose of this study was to examine relationships between neighborhoods and internalizing symptoms in the study population.	Youth N = 775	Higher social cohesion is associated with decreasing anxiety and depression symptoms.	N/A
[73] 2020 United States	Neighborhood profiles and associations with coping behaviors among low-income youth	Cross-sectional	The purpose of this study was to identify whether youths’ engagement in coping behaviors was related to profile membership.	African–American youth N = 733	The four neighborhood profiles identified: Highest disorder Highest violence/highest disadvantage High violence Highest cohesion/lowest disorder	Increased protective factors ---> more resilience Increased risk factors ---> less resilience
[74] 2008 United States	Neighborhood disorganization, substance use, and violence among adolescents in Puerto Rico	Cross-sectional	The purpose of this study was to examine social disorder and youth violence.	Parents and adolescents N = 691	Youth violence associated with social disorder.	Risk factors: Social disorder Hearing gunfire and open-carry weapons Witnessing activities related to theft Vandalism Using alcohol and drugs
[75] 2019 United States	A longitudinal investigation of protective factors for bereaved, maltreated youth	Cohort study	The purpose of this study was to examine the effects of bereavement on internalizing and externalizing psychopathology.	Youth and caregivers N = 800	Compared to non-bereaved maltreated youth, maltreated, bereaved youths were at risk of externalizing symptoms.	Protective factors: Individual and family future orientation Parental monitoring Neighborhood collective efficacy
[76] 1997 United States	“Missed, dissed, and pissed”: making meaning of neighborhood risk, fear, and anger management in urban black youth	Cross-sectional	The purpose of this study was to test the association between risk factors and stress and coping methods in the study population.	African–American adolescents N = 202	High calamity fears diminished the anger response of the study population.	Protective factor process:Kinship social support —> anger suppression for youth in high-risk environments
[77] 2006 United States	Neighborhood risk and the development of resilience	Cross-sectional	The purpose of this study was to examine protective factors associated with positive adjustment among the study population.	Male youth N = 310	Protective factors measured in early childhood predicted positive adjustment at ages 11 and 12 years.	Protective factors: Parent–child relationship quality
[78] 2020 United States	Long-term neighborhood effects on adolescent outcomes: mediated through adverse childhood experiences and parenting stress	Cohort study	The purpose of this study was to examine childhood experiences and adolescent anxiety.	Male children and mothers N = 4898	Significant mediators: Mothers‘ parenting stress Exposure to ACEs Neighborhood-concentrated poverty only exerted indirect relations.	Protective factors: Collective efficacy Healthy family
[79] 2013 Jamaica	The new imperative: reducing adolescent-related violence by building resilient adolescents	Literature review	The purpose of this study was to explore the literature on the impact of violence on child behavior.	Children N/A	Programmatic areas needed for violence prevention: Family connectedness Educational enrichment Economic opportunities	Risk factors: Childhood violence
[80] 2021 United States	Socio-ecological predictors of resilience development over time among youth with a history of maltreatment	Cohort study	The purpose of this study was to identify socio-ecological predictors of resiliency changes over time among the study population.	Adolescents N = 771	Resiliency over time was associated with (a) younger age; (b) high quality of parent–child relationship; and (c) neighborhood safety.	Risk Factors: Violence (inhibits resilience)

Note: N/A = not applicable.

## Data Availability

Not applicable.

## Supplementary Materials

The following supporting information can be downloaded at: https://www.mdpi.com/article/10.3390/children10111791/s1, Table S1. Conceptualization of Resilience and Levels of Resilience.
