# Supplementary material for: Flexible Resources Key to Neighborhood Resilience for Children: A Scoping Review
Source: Children (Basel). 2023 Nov 7;10(11):1791. doi: 10.3390/children10111791 (PMC10670030; doi:10.3390/children10111791)
Supplement: Supplementary file 1 [file children-10-01791-s001.zip › children-2664366-supplementary.pdf]

Supplementary Table S1. Conceptualization of Resilience and Levels of Resilience.

Author<br>Year of Publication<br>Country	Conceptualization of Resilience	Level of Resilience
[24]<br>2008<br>United States	Resilience was conceptualized as the collective attributes that protect citizens from poor outcomes.	Neighborhood
[25]<br>2008<br>United States	Resilience was conceptualized as individual youth attributes to overcome adversity.	Individual
[26]<br>2018<br>United States	Resilience was conceptualized as individual ability to “bounce back” post adversity.	Individual
[27]<br>2016<br>United States	Resilience was conceptualized as parenting attributes that mitigate neighborhood disadvantage.	Family
[28]<br>2022<br>United States	Resilience was conceptualized as a family’s adaptation to stressors.	Individual, Family, and Neighborhood
[29]<br>2021<br>United States	Resilience was conceptualized as “inoculating effects” of stress among traumatized youth.	Individual
[30]<br>2022<br>United States	Resilience was conceptualized as successful family practices that allow families to adapt to setbacks.	Family
[31]<br>2021<br>United States	Resilience was conceptualized as individual flourishing and self-regulation, Additionally, family resilience was conceptualized as family and neighborhood supports that promote youth resilience.	Individual and Family
[32]<br>2018<br>United States	Resilience was conceptualized as neighborhood attributes (e.g., safety, trust, engagement, leadership, and representation) within a neighborhood that increase environmental health adaption.	Neighborhood
[33]<br>2013<br>United States	Resilience was conceptualized as a youth’s ability to achieve educational attainment in spite of neighborhood disadvantage.	Individual
[34]<br>2015<br>United States	Resilience was conceptualized as neighborhood social resources that promote youth’s adaption to trauma.	Individual and Neighborhood
[35]<br>2012<br>United States	Resilience was conceptualized as an adaptive process that promotes capacities to respond and recover from weather related disasters.	Neighborhood

[36] 2019 United States	Resilience was conceptualized as family attributes such as stable relationship with children that promote the youth's ability to adapt to adversities.	Individual and Family
[37] 2021 United States	Resilience was conceptualized as family attributes such as stable relationship with children that promote the youth's ability to adapt to adversities.	Individual and Family
[38] 2022 South Africa	Resilience was conceptualized as healthy youth development as measured by growth, cognition, and behavior.	Individual and Family
[39] 2015 Australia	Resilience was conceptualized as healthy youth weight and body mass index.	Individual
[40] 2021 United States	Resilience was conceptualized as successful family practices that allow families to adapt to setbacks.	Individual and Family
[41] 2022 United States	Resilience was conceptualized as successful family practices that allow families to adapt to setbacks. Childhood resilience was defined as being protected from increased risk of obesity.	Individual, Family, and Neighborhood
[42] 2018 United Kingdom	Resilience was conceptualized as individual, family, and neighborhood attributes that protect poly-victims against risks of psychosis.	Individual, Family, and Neighborhood
[43] 2019 United States	Resilience was conceptualized as healthy adaptive skills for youth.	Individual
[44] 2000 United States	Resilience was conceptualized as attributes that promoted adaptation to stressors.	Individual
[45] 2009 United States	Resilience was conceptualized as neighborhood attributes that promote wellbeing of at risk youth.	Neighborhood
[46] 2020 United States	Resilience was conceptualized as strategies youth can use to survive adverse neighborhood environments.	Individual and Neighborhood
[47] 2020 United States	Resilience was conceptualized as protective factors that promoted adaptation to stress.	Individual
[48] 2016 Cali, Columbia	Resilience was conceptualized as capacity to anticipate and mitigate infection.	Neighborhood
[49] 2018 United States	Resilience was conceptualized as mitigation of poor outcomes and promotion of healthy outcomes after adversity.	Individual
[50] 2021 United States	Resilience was conceptualized as capacity to mitigate risk after adversity.	Individual and Neighborhood
[51] 2022 United States	Resilience was conceptualized as youth maintaining activity during COVID-19	Individual
[52] 2018 United States	Resilience was conceptualized as successful family practices that allow families to adapt to setbacks. Youth resilience was conceptualized as demonstrated control and calmness in face of adversity.	Family and Individual

[53]<br>1999<br>United States	Resilience was conceptualized as capacity to live a healthy life despite environmental adversity.	Individual
[54]<br>2018<br>United States	Resilience was conceptualized as family and neighborhood supports that promote adaptation despite risk.	Individual, Family, and Neighborhood
[55]<br>2018<br>United States	Resilience was conceptualized as individual, family, and neighborhood supports that promote academic functioning despite maltreatment.	Individual, Family, and Neighborhood
[56]<br>2014<br>United States	Resilience was conceptualized as individual, family, and neighborhood supports that promote academic success and adherence to behavior norms.	Individual, Family, and Neighborhood
[57]<br>2020<br>United States	Resilience was conceptualized as individual, family, and neighborhood attributes that promote wellbeing among adolescents from families who have encountered the justice system.	Individual, Family, and Neighborhood
[58]<br>2018<br>United States	Resilience was conceptualized as thriving despite socioeconomic disadvantages.	Individual
[59]<br>2004<br>United States	Resilience was conceptualized as protective factors that promoted health adaptation to asthma.	Individual
[60]<br>2021<br>United States	Resilience was conceptualized as neighborhood protective factors that mitigate the risk factors of neighborhood disorder regarding initial adolescent substance use.	Individual and Neighborhood
[61]<br>1994<br>United States	Resilience was conceptualized as individual and neighborhood attributes that promote individuals making healthy choices.	Individual and Neighborhood
[62]<br>2021<br>United States	Resilience was conceptualized as individual, family, and neighborhood attributes that mitigate risks from adverse childhood experiences (ACEs).	Individual, Family, and Neighborhood
[63]<br>2018<br>United States	Resilience was conceptualized as neighborhood and family protections that reduce risks of childhood overweight/obesity incidence.	Individual, Family, and Neighborhood
[64]<br>2022<br>United States	Resilience was conceptualized as neighborhood and family protections that promote adaption for at-risk families and children.	Individual, Family, and Neighborhood
[65]<br>2007<br>United States	Resilience was conceptualized as the interplay between the risk and protective factors that influence individual health.	Individual
[66]<br>2021<br>United States	Resilience was conceptualized as neighborhood and parental protective attributes that promoted reduced exposure/infection and worry for children during the COVID-19 pandemic.	Individual, Family, and Neighborhood
[67]<br>2021<br>United States	Resilience was conceptualized as neighborhood and family protections that promote youth to succeed in school and avoid the justice system,	Individual, Family, and Neighborhood
[68]<br>2021<br>United States	Resilience was conceptualized as the neural mechanisms that promote adaptation against neighborhood violence.	Individual

[69]<br>1999<br>United States	Resilience was conceptualized as the protective factors that promoted school success despite neighborhood disorder.	Individual
[70]<br>2021<br>United States	Resilience was conceptualized as successful family practices that promote healthy behaviors of children.	Individual and Family
[71]<br>2022<br>United States	Resilience was conceptualized as neighborhood and family protections that mitigate risk of school discipline and police contact for children.	Individual, Family, and Neighborhood
[72]<br>2016<br>United States	Resilience was conceptualized as neighborhood factors that protect against childhood temperamental withdrawal.	Individual and Neighborhood
[73]<br>2020<br>United States	Resilience was conceptualized as individual and neighborhood attributes that mitigate risk of neighborhood disorder for children.	Individual and Neighborhood
[74]<br>2008<br>United States	Resilience was conceptualized as protective factors that mitigate risk of childhood violence related to exposure of neighborhood disorder.	Individual, Family, and Neighborhood
[75]<br>2019<br>United States	Resilience was conceptualized as neighborhood and family supports that promote adaption for bereaved maltreated youth.	Individual, Family, and Neighborhood
[76]<br>1997<br>United States	Resilience was conceptualized as neighborhood supports that mitigate the risk of neighborhood disorder and promote adaptation to stress.	Individual and Neighborhood
[77]<br>2006<br>United States	Resilience was conceptualized as neighborhood and family supports, and individual attributes that mitigate adverse outcome when children have adverse experiences.	Individual, Family, and Neighborhood
[78]<br>2020<br>United States	Resilience was conceptualized as neighborhood-built environment attributes that promote increased physical activity during the COVID-19 pandemic.	Individual and Neighborhood
[79]<br>2013<br>Jamaica	Resilience was conceptualized as environmental supports that protect adolescences from disadvantage.	Individual, Family, and Neighborhood
[80]<br>2021<br>United States	Resilience was conceptualized as neighborhood, family, and individual attributes that promote adolescent adaption.	Individual, Family, and Neighborhood
